# CDC and YMCA: A Promising Partnership for Delivering Fall Prevention Programing

**DOI:** 10.3389/fpubh.2014.00235

**Published:** 2015-04-27

**Authors:** Heidi Ehrenreich, Maureen Pike, Katherine Hohman, Margaret Kaniewski, Matt Longjohn, Gaya Myers, Robin Lee

**Affiliations:** ^1^Division of Unintentional Injury Prevention, National Center for Injury Prevention and Control, Centers for Disease Control and Prevention, Atlanta, GA, USA; ^2^YMCA of the USA, Chicago, IL, USA

**Keywords:** fall prevention, Tai Chi, community-based programs, partnerships, dissemination

## Older Adult Falls

Falls threaten the independence of adults aged 65 years and older. In the U.S., one in three older adults fall annually, causing significant disability and reduced quality of life (1). The high prevalence of falls, coupled with more than $30 billion in direct medical costs (2) has created a critical need for effective older adult fall prevention programs. As the nation’s public health agency, the Centers for Disease Control and Prevention (CDC) is committed to identifying ways to reduce the burden of older adult falls. In this commentary, we describe a promising approach to reach older adults with effective interventions by partnering with the YMCA to deliver community-based fall prevention programs.

## Implementation of Effective Community Fall Prevention Programs

Centers for Disease Control and Prevention has produced several guides dedicated to fall prevention programing and delivery. The *CDC Compendium of Effective Fall Interventions* is intended to help public health practitioners use the best scientific evidence to effectively address falls among older adults in the community (3).

The Compendium describes 22 scientifically tested interventions for use by public health practitioners, aging service providers, and others. In addition, CDC developed a *how-to* guide for community-based organizations seeking to develop, implement, and evaluate their own effective fall prevention programs (4).

While federal and state public health agencies have used CDC’s guide to implement fall prevention programs, the information about effective programs does not always reach the intended audience (5). This is largely due to the lack of local infrastructure needed to deliver community-based programs (6). Developing and maintaining the necessary organizational infrastructure can be time consuming and costly, limiting program sustainability. A recent systematic review revealed that stable financial program support, integrated programing, and the ability to make program adaptations were major factors that sustained successful fall prevention programs (7).

YMCA’s robust infrastructure for program delivery, large membership base, and local credibility offer strong potential for building successful and wide-reaching public health programing. The marketing literature supports the use of these types of distribution channels to improve the adoption and implementation of evidence-based programs (8). For these reasons, CDC is pursuing partnerships with organizations such as YMCAs to help implement effective fall prevention programs.

## Partnering with the YMCA to Scale-up Successful Public Health Programs

YMCAs are independent but federated organizations working to spread health and wellness in their communities. YMCAs offer classes for all ages, all skill levels, and all interests. As a national resource center, YMCA of the USA (Y-USA) supports over 2,600 YMCAs located across 10,000 U.S. neighborhoods and with 20.6 million members (http://www.ymca.net).

Y-USA has a history of collaborating on national public health initiatives (9, 10). Y-USA partnered with CDC and the National Association of Chronic Disease Directors to disseminate *EnhanceFitness*, an evidence-based exercise program for older adults. In the program, trained YMCA fitness staff and volunteers lead a comprehensive exercise routine shown to increase physical, mental, and social functioning in older adults (11). In the first year of this partnership, the program reached 2,000 older adults at 41 YMCAs. In another initiative, 157 YMCAs partnered with the LIVESTRONG Foundation for extensive training as hubs of support for cancer survivors. To date, over 21,000 survivors have been served by this initiative. Finally, CDC partnered with Y-USA to reach over 19,000 participants at 128 YMCAs to expand its evidence-based National Diabetes Prevention Program to participating communities (12). As part of this CDC-led program, YMCAs have trained their wellness instructors as “lifestyle coaches” to implement lifestyle-change programs focused on participants losing weight, being physically active, and coping with stress.

## YMCA Adapts CDC’s Older Adult Fall Prevention Program

Motivated by the success of the National Diabetes Prevention Program, CDC initiated a similar strategy to implement an evidence-based older adult fall prevention program using the YMCA infrastructure. With funding from the CDC, Y-USA licensed the rights to the *Tai Chi Moving for Better Balance* fall prevention program (13) and adapted the program to fit the YMCA training system. Y-USA reintroduced the program under the name, *Y-Moving for Better Balance* (Y-MFBB) and contracted with the program’s creator to train YMCA Faculty Trainers as Y-MFBB instructors. As of September 2013, 287 Y-MFBB instructors have been trained. To encourage implementation of Y-MFBB locally, Y-USA awarded 131 YMCAs grants of $1000 each to hold instructor trainings and initiate Y-MFBB classes.

**Figure 1:**
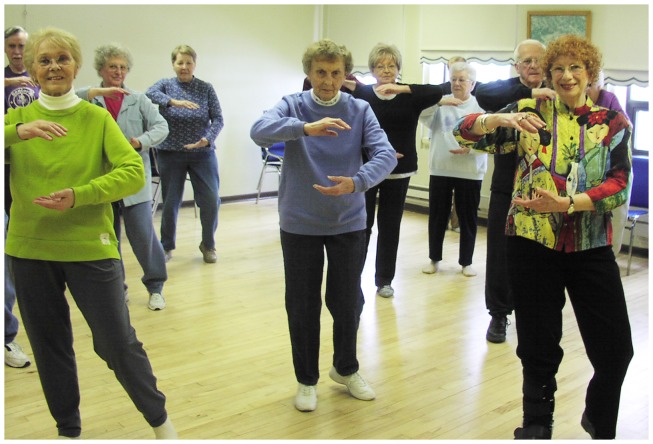
**Y-MFBB Class in Broome County, New York**.

In fall 2013, the Y-USA interviewed staff directors from 8 of the 75 YMCAs offering Y-MFBB in the last year to gather lessons learned about local Y-MFBB implementation. After approximately 17 months, these 8 YMCAs had reached 706 participants. Most participants were women, aged 65 years and older, and YMCA members (see Figure 1 depicting Y-MFBB participants). Participants reported discovering the Y-MFBB program through YMCA advertising, local community organizations, and from medical professionals. Cost to participate ranged from no additional charge for members to $70 per 12-week session, or approximately $3 per class, for a YMCA offering the program at an off-site facility. Four YMCAs reported an implementation cost of $386 per 12-week session, based mainly on instructor time.

Overall, staff directors determined that fall prevention programing fit well with YMCA’s health and wellness mission, citing two main reasons for offering Y-MFBB: (1) the growing number of older adult members; and (2) the high incidence of falls among them. However, Y-MFBB instructors also need the opportunity to check program fidelity and offer progressively more challenging classes. With more Y-MFBB instructors and training, the directors are considering placing Y-MFBB into a larger portfolio of falls prevention programing at their sites. CDC is currently supporting the development of a national Y-MFBB rollout plan based on further research into program and implementation effectiveness.

## Next Steps

While CDC and state public health agencies have the tools to assess and address public health problems, community-based organizations are often tasked with delivering programs. The YMCA has been an important partner for scaling up the CDC fall prevention program to a wider and more diverse audience. This example of leveraging partnerships with organizations that already have a robust infrastructure in place for large-scale program delivery is critical for population-level gains. CDC will continue to work with organizations such as the YMCA to increase the availability of Y-MFBB and other evidence-based fall prevention programs to reduce fall risk among older adults.

## Conflict of Interest Statement

The authors declare that the research was conducted in the absence of any commercial or financial relationships that could be construed as a potential conflict of interest.

This paper is included in the Research Topic, “Evidence-Based Programming for Older Adults.” This Research Topic received partial funding from multiple government and private organizations/agencies; however, the views, findings, and conclusions in these articles are those of the authors and do not necessarily represent the official position of these organizations/agencies. All papers published in the Research Topic received peer review from members of the Frontiers in Public Health (Public Health Education and Promotion section) panel of Review Editors. Because this Research Topic represents work closely associated with a nationwide evidence-based movement in the US, many of the authors and/or Review Editors may have worked together previously in some fashion. Review Editors were purposively selected based on their expertise with evaluation and/or evidence-based programming for older adults. Review Editors were independent of named authors on any given article published in this volume.
